# The effect of remote ischaemic preconditioning on postoperative cardiac and inflammatory biomarkers in pancreatic surgery: a randomized controlled trial

**DOI:** 10.1093/bjsopen/zrab015

**Published:** 2021-04-24

**Authors:** L van Zeggeren, R A Visser, L M Vernooij, I M Dijkstra, M Bosma, Q Molenaar, H C van Santvoort, P G Noordzij

**Affiliations:** Departments of Anaesthesiology, Intensive Care and Pain Medicine and Clinical Chemistry; Departments of Anaesthesiology, Intensive Care and Pain Medicine and Clinical Chemistry; Departments of Anaesthesiology, Intensive Care and Pain Medicine and Clinical Chemistry; Department of Anaesthesiology, Intensive Care and Emergency Medicine, University Medical Centre Utrecht, Utrecht, The Netherlands; St. Antonius Hospital, Nieuwegein, the Netherlands; St. Antonius Hospital, Nieuwegein, the Netherlands; Regional Academic Cancer Centre Utrecht, Department of Hepato-Pancreato-Biliary Surgery, St. Antonius Hospital Nieuwegein & University Medical Centre Utrecht, the Netherlands; Regional Academic Cancer Centre Utrecht, Department of Hepato-Pancreato-Biliary Surgery, St. Antonius Hospital Nieuwegein & University Medical Centre Utrecht, the Netherlands; Departments of Anaesthesiology, Intensive Care and Pain Medicine and Clinical Chemistry

## Abstract

**Background:**

Cardiac and inflammatory biomarkers have been associated with adverse outcome after major abdominal surgery. This study investigated the effect of remote ischaemic preconditioning (RIPC) on perioperative concentrations of high-sensitive cardiac troponin (hs-cTn) T and interleukin (IL) 6.

**Methods:**

Adult patients scheduled for elective pancreatic surgery between March 2017 and February 2019 were randomized to either three cycles of upper-limb ischaemia and reperfusion (each 5 min) or a sham procedure before surgery. The primary endpoint was the maximum postoperative hs-cTnT concentration within 48 h after surgery. Secondary endpoints were postoperative myocardial injury (PMI), defined as an absolute increase of hs-cTnT of at least 14 ng/l above baseline concentration, maximum concentration of IL-6 within 48 h after surgery and postoperative complications within 30 days of surgery.

**Results:**

Of 99 eligible patients, 46 underwent RIPC and 46 a sham procedure. RIPC did not reduce the maximum hs-cTnT concentration after surgery (12.6 ng/l RIPC, 16.6 ng/l controls, *P* = 0.225), nor did it lessen the incidence of PMI (15/45 RIPC, 18/45 controls, *P* = 0.375). The maximum postoperative IL-6 concentration was 265 pg/ml after RIPC versus 385 pg/ml in controls (*P* = 0.108). Postoperative complications occurred in 23 RIPC and 24 control patients respectively.

**Conclusions:**

Remote ischaemic preconditioning did not reduce the maximum postoperative hs-cTnT concentration. Postoperative myocardial injury, IL-6 concentrations and postoperative complications were similar between RIPC patients and controls.

**Trial registration:**

Clinicaltrials.gov identifier NCT03460938.

## Introduction

Despite improvements in perioperative care, complication rates after major non-cardiac surgery remain substantial^1–3^ and are important determinants of poor functional recovery and long-term survival^2^^,^^4^^,^^5^. Although the aetiology of postoperative complications is not fully understood, myocardial injury has consistently been associated with poor outcomes after non-cardiac surgery, including major abdominal surgery, in a concentration-dependent manner^1^^,^^6^.

Besides ischaemia, other pathophysiological mechanisms are related to postoperative myocardial injury (PMI). Surgery can trigger a profound inflammatory response, characterized by high circulating levels of pro-inflammatory cytokines. Endothelium-derived nitric oxide overproduction induces tissue hypoperfusion and mitochondrial dysfunction. The reperfusion injury that follows is recognized as an important cause of subsequent organ dysfunction^7^^,^^8^. High levels of inflammation in patients undergoing major abdominal surgery have previously been associated with PMI and postoperative complications^9^^,^^10^, but no treatments have been proven to protect organs from ischaemia–reperfusion injury due to inflammation during or shortly after non-cardiac surgery.

Ischaemic preconditioning is a physiological mechanism that uses brief cycles of ischaemia and reperfusion to protect organs from further ischaemic insults. Studies in cardiac surgery patients have demonstrated a cardioprotective effect of remote ischaemic preconditioning (RIPC), although not in postoperative outcome^11–13^. The effect of RIPC on cardiac and inflammatory biomarkers in abdominal surgery is largely unknown. This study aimed to investigate the effects of RIPC on peak concentrations of high-sensitive cardiac troponin T (hs-cTnT) and interleukin (IL) 6 in patients undergoing pancreatic resection.

## Methods

### Trial design

The myocardial injury and complications after major abdominal surgery (MICOLON) 2 study was an investigator-initiated, single-centre, randomized, double-blinded controlled trial. The study was conducted at St. Antonius Hospital, Nieuwegein of the Regional Academic Cancer Centre Utrecht, a tertiary referral hospital for pancreatic surgery in the Netherlands with an annual volume of over 100 pancreatoduodenectomies. The study protocol was approved by the Medical Research Ethics Committees United (MEC-U, number R16.042) and registered at clinicaltrials.gov (NCT03460938). The study was performed in accordance with the principles of the Declaration of Helsinki. All patients provided written informed consent. This study was performed and reported according to the CONSORT guidelines for RCTs^14^.

### Patients

Adult patients aged 18 years or older scheduled for elective pancreatic surgery (pancreatoduodenectomy, distal pancreatectomy and total pancreatectomy) between March 2017 and February 2019 were eligible for study participation. There were no exclusion criteria apart from pregnancy. All patients underwent routine preoperative assessment for co-morbidities at an outpatient preoperative anaesthesia clinic.

Patients and the public were not involved in the design, conduct, reporting or dissemination plans of this research.

### Trial procedures and blinding

Patients received oral and written study information at the anaesthesia outpatient clinic and, when willing to participate, provided signed informed consent on the day before surgery. Participants were randomly assigned on the day of surgery to the RIPC group or control group in a 1 : 1 ratio, with no stratification for age, co-morbidities, histology or proposed operation. Randomization was performed using a web-based system, in permuted blocks, with variable block sizes. RIPC was applied after induction of anaesthesia and before incision by an appointed anaesthesiologist, who was aware of the study-group assignment. Individual patients, surgeons and the investigators, who obtained and documented study data and clinical endpoints, were blinded to study group assignment. The RIPC protocol consisted of three 5-min cycles of upper-limb ischaemia, induced by an automated cuff-inflator placed on the upper arm and inflated to 200 mmHg, with an intervening 5 min of reperfusion when the cuff was deflated. A sham procedure was performed in control patients, that consisted of a deflated cuff placed on the upper limb for 30 min.

### Perioperative care and blood sample analysis

Surgery was performed under general anaesthesia and epidural analgesia, unless a contraindication for epidural analgesia existed. To avoid interaction with ischaemic preconditioning, propofol was not used for induction or maintenance of anaesthesia. Instead, sodium thiopental (Eureco Pharma, Ridderkerk, The Netherlands) or midazolam (Accord Healthcare, Utrecht, The Netherlands) were used for induction of anaesthesia and sevoflurane (Sevorane^®^, Abbvie, Hoofddorp, The Netherlands) for maintenance. Further anaesthetic management was at the discretion of the attending anaesthesiologist. All patients had an arterial line and central venous catheter for haemodynamic monitoring. Duration of surgery, fluid balance, blood loss, transfusion of blood products and use of inotropes or vasopressors were recorded. After surgery, all patients were routinely admitted to an intensive care unit (ICU) for the first postoperative day and managed according to the Enhanced Recovery After Surgery (ERAS) guidelines^15^. Discharge from the ICU was based on standard operating procedures at the discretion of the attending intensivist.

Blood samples were collected for the measurement of hs-cTnT and IL-6 concentrations after induction of anaesthesia, before surgical incision (baseline) and at 4, 12, 24 and 48 h. Blood samples were frozen and stored at -80°C until batch analysis. Analyses of both hs-cTnT and IL-6 were performed on an automated Cobas^®^ 8000 platform (Roche Diagnostics, Mannheim, Germany). Hs-cTnT analysis was done using a fifth generation Elecsys^®^ Troponin T high-sensitivity assay, IL-6 analysis was performed using the Elecsys^®^ IL-6 assay (Roche Diagnostics, Mannheim, Germany).

During the postoperative period, clinical data including postoperative complications were registered in the electronic patient record, as a part of standard care. Study data were entered in the REDCap^®^ (Vanderbilt University, Nashville, United States) database management system by investigators blinded for treatment allocation.

### Outcomes

The primary endpoint was the maximum postoperative hs-cTnT concentration within 48 h after surgery. Secondary endpoints were the maximum postoperative concentration of IL-6 within 48 h after surgery, PMI and postoperative complications within 30-days of surgery. PMI was defined as an absolute increase of hs-cTnT of at least 14 ng/l above baseline concentration. A cut-off value for an elevated IL-6 concentration was set at 432 pg/ml based on prior research^9^. Postoperative complications were graded according to the Clavien-Dindo (CD) classification of surgical complications and the Comprehensive Complication Index (CCI)^16^^,^^17^. Pancreatic-specific complications (i.e., pancreatic fistula, bile leakage, postpancreatectomy haemorrhage, delayed gastric emptying, chyle leakage) were defined and classified according to the International Study Group of Pancreatic Surgery (ISGPS) definitions^18–22^. Only grade B and C have been reported as these are generally considered clinically relevant.

### Statistical analysis

Based on prior study at this institution on the association between postoperative hs-cTnT levels and complications in patients undergoing pancreatic resection (mean(s.d.) peak postoperative hs-cTnT 24(20) ng/l) it was hypothesized that two groups of 45 patients were required to yield an 80 per cent power and a significance level of 0.05, to demonstrate a 50 per cent reduction in peak hs-cTnT concentration compared to the control group, on the basis that such a reduction would result in a cardiac troponin (cTn) concentration of less than 0.014 ng/l, the diagnostic cut off for the cTn assay^1^.

Patients were analysed based on an intention-to-treat principle. Baseline characteristics were described per treatment arm as percentages, mean(s.d.), or median (i.q.r.) as appropriate. Baseline differences between treatment arms were assessed, as were requirement for red blood cell (RBC) transfusion and duration of surgery. Multivariable analyses were used to adjust for this imbalance. For the primary outcome, linear regression was performed to compare mean peak postoperative hs-cTnT concentrations between both groups. Before analysis, hs-cTnT was log transformed and back-transformed geometric mean hs-cTnT concentrations were reported for both groups with 95 per cent confidence intervals. Generalized linear mixed models were used to analyse the effect of RIPC on postoperative hs-cTnT concentrations over time (i.e., at 4, 12, 24 and 48 h after surgery). Univariable analyses were performed with time and treatment group as fixed parts and ‘subject’ as random part. To assess whether postoperative hs-cTnT measurements differed over time between both groups, an interaction term between treatment group and time was added to the fixed part of the model. Multivariable analysis was then conducted with adjustments for preoperative hs-cTnT, RBC transfusion and duration of surgery. Similar analyses were performed for the secondary outcomes. As IL-6 trajectories were not linear over time, B-splines were applied using three knots for time to improve model fit. Models were compared based on the Akaike’s information criterion. Restricted maximum likelihood estimation was used to generate unbiased variance estimates for the final models.

Clinical outcomes, including postoperative complications, PMI and elevated IL-6, were compared using a χ^2^ test, independent samples T test or Mann–Whitney U test, as appropriate. Two-sided *P* values of 0.050 or less were considered statistically significant. Data were analysed using SPPS versions 24–26 (IBM, Amsterdam, The Netherlands) and R statistics version 3.5.1 (R, Inc., Boston, United States).

## Results

### Population

**Fig. 1:**
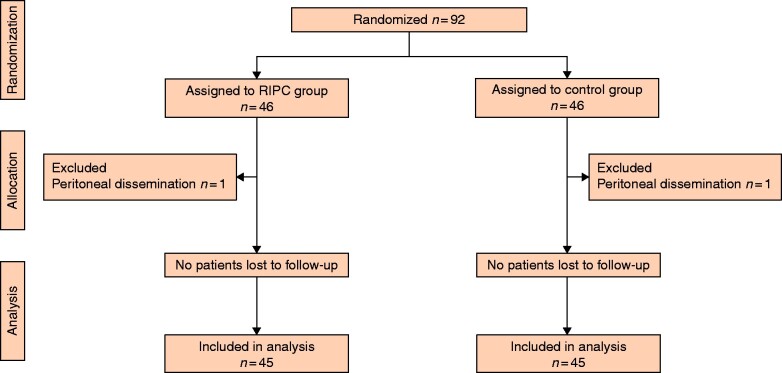
CONSORT diagram RIPC, remote ischaemic preconditioning.

Of 99 patients considered eligible, 92 were randomized, of whom 90 were included in the final analysis (45 RIPC, 45 control). In two patients, surgery was cancelled due to peritoneal dissemination (*Fig. 1*). Median age was 69 years and 59 per cent of patients were male. A history of coronary artery disease (i.e., myocardial infarction or coronary revascularization) was present in 20 per cent of patients (*Table 1*). The majority of patients had surgery for pancreatic cancer, with postoperative epidural analgesia. None of the patients were lost to follow-up and no RIPC-related adverse events were observed. Both groups were well matched, except for differences in numbers who received RBC transfusions and the duration of surgery, both of which were greater in controls.

**Table 1:** Baseline characteristics

Characteristic	RIPC (*n* = 45)*	Control (*n* = 45)*
**Age (years)^†^**	69 (68–72)	69 (65–73)
**Male sex**	26 (58)	27 (60)
**Prior diagnosis**		
Hypertension	17 (38)	17 (38)
Atrial fibrillation	9 (20)	6 (13)
Myocardial infarction	8 (18)	3 (7)
Coronary revascularization	8 (18)	6 (13)
Cardiac failure	3 (7)	2 (4)
Diabetes mellitus	8 (18)	15 (33)
Stroke	1 (2)	4 (9)
COPD	5 (11)	4 (9)
Renal insufficiency	4 (9)	6 (13)
Peripheral artery disease	5 (11)	4 (9)
**ASA classification**		
I	1 (2)	5 (11)
II	23 (51)	23 (51)
III	19 (42)	15 (33)
IV	2 (4)	2 (4)
**Medications**		
Sulfonylureas	4 (9)	7 (16)
Angiotensin receptor blockers	6 (13)	4 (9)
Nitrates	3 (7)	3 (7)
Aspirin	10 (22)	6 (13)
**Type of surgery**		
Pancreatoduodenectomy	35 (78)	39 (87)
Distal pancreatectomy (± splenectomy)	9 (20)	3 (7)
Total pancreatectomy	1 (2)	3 (7)
**Cancer surgery**	39 (87)	43 (96)
**Robot-assisted surgery**	19 (42)	15 (33)
Conversion to open procedure	2 (4)	4 (9)
**Epidural catheter**	39 (87)	38 (84)
**Operating time (min)^‡^**	329 (106)	376 (110)
**Blood loss (ml)^†^**	400 (0–5100)	400 (0–7000)
**Intraoperative fluid balance (ml)^†^**	2030 (150–11 000)	2650 (-1500–8000)
**RBC transfusion**	3 (7)	10 (22)
**Use of inotropes and/or vasopressors**	42 (93)	39 (87)

* Values in parentheses are percentages unless indicated otherwise;

† values are median (i.q.r.),

‡ values are mean(s.d.). COPD, chronic obstructive pulmonary disease; RBC, red blood cell.

### Cardiac biomarkers

**Fig. 2:**
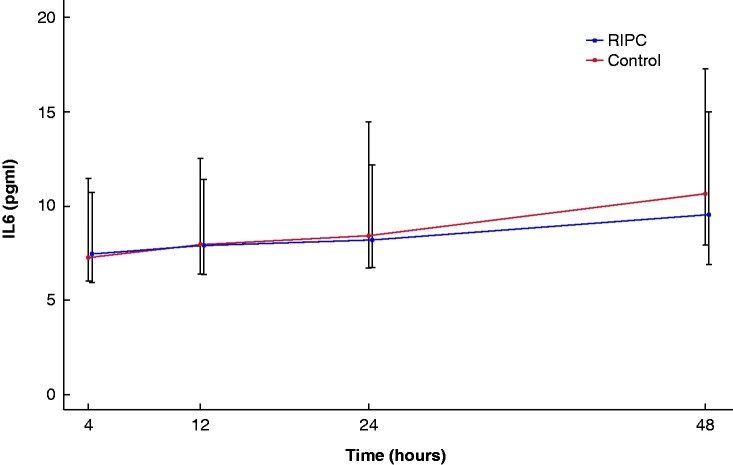
Postoperative high-sensitive cardiac troponin concentrations over time in the remote ischaemic preconditioning (RIPC) group and control group Estimates are presented from the generalized linear mixed model, adjusted for preoperative high-sensitive cardiac troponin T (hs-cTnT), red blood cell transfusion and duration of surgery. The 25^th^ and 75^th^ percentiles are presented around each of the hs-cTnT measurements. Nine (2%) hs-cTnT samples were missing.

Median preoperative hs-cTnT concentration was 9 (i.q.r. 5–12) ng/l and was similar in the two groups (*P* = 0.564). Before surgery, hs-cTnT was elevated (i.e. hs-cTnT at least 14 ng/l) in 9/45 patients in the RIPC group and 10/45 in the control group (*P* = 0.434). RIPC did not reduce the maximum hs-cTnT concentration after surgery (RIPC 12.6 (95 per cent c.i. 5.9 to 27.0) ng/l, controls 16.6 (95 per cent c.i. 12.1 to 22.8) ng/l, *P* = 0.225), nor did it lessen the incidence of PMI (RIPC 13 patients, controls 18 patients, *P* = 0.375). Profiles for postoperative hs-cTnT concentrations were similar between both groups with time (*P* = 0.197) (*Fig. 2*).

### Inflammatory biomarkers

**Fig. 3:**
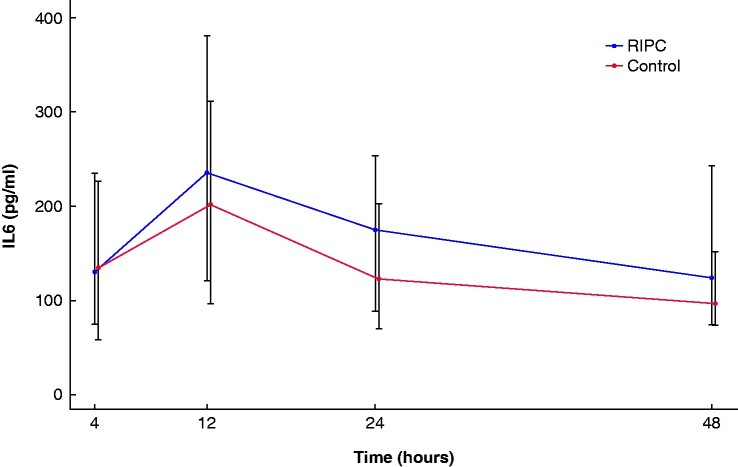
Postoperative interleukin (IL) 6 concentrations over time in patients in the remote ischaemic preconditioning (RIPC) group and control group Estimates are presented from the generalized linear mixed model, adjusted for preoperative IL-6, red blood cell transfusion and duration of surgery. The 25^th^ and 75^th^ percentiles are presented for each time interval. Nine (2%) IL-6 samples were missing.

Median preoperative IL-6 concentration was 4.4 (i.q.r. 3–7.7) pg/ml and did not differ between groups (*P* = 0.930). The maximum postoperative IL-6 concentration was also the same in both groups (RIPC 239 (i.q.r. 115–360) pg/ml, controls 317 (i.q.r. 174–909) pg/ml, *P* = 0.134). There was no reduction in maximum absolute increase in IL-6 concentration, compared to baseline following RIPC (265 (95 per cent c.i. 122 to 1565) pg/ml RIPC, 385 (95 per cent c.i. 280 to 531) pg/ml controls, *P* = 0.108). Postoperative IL-6 concentration greater than 432 pg/ml was found in 13 RIPC patients and 16 controls (*P *= 0.148). Over time, there were no differences in postoperative IL-6 concentrations between groups (*P* = 0.587) (*Fig. 3*).

### Clinical outcomes

Overall median blood loss was 400 ml and 14 per cent of patients received an RBC transfusion (3 RIPC, 10 controls). Incidences of postoperative complications are presented in *Table 2*. A postoperative complication, defined as a CD class 3 or above or ISGPS class grade B or C, occurred in 23/45 RIPC patients, and 24/45 controls (*P* = 1.000). Median CCI was 27.6 (i.q.r. 20.9–35.9) following RIPC and 30.8 (i.q.r. 16.6–47.7) in control patients (*P* = 0.494). Mean(s.d.) duration of ICU stay (RIPC 1.8(0.2) days, controls 2.7(0.4) days; *P* = 0.065), overall duration of hospital stay (RIPC 16.5(1.9) days, controls 19(2.5) days, (*P* = 0.353) and readmission rates within 30 days (RIPC 9/45, controls 7/45, *P* = 0.581) also did not differ.

**Table 2:** Clinical outcomes

Postoperative complication	RIPC (*n* = 45)	Control (*n* = 45)	*P*
**Death**	0	1 (2)	1.000
**Pancreatic fistula**	12 (27)	11 (24)	1.000
**Bile leakage**	2 (4)	3 (7)	1.000
**Post-pancreatectomy haemorrhage**	1 (2)	3 (7)	0.609
**Delayed gastric emptying**	10 (22)	9 (20)	1.000
**Chyle leakage**	1 (2)	7 (16)	0.064
**Myocardial infarction**	0	0	1.000
**Stroke**	1 (2)	1 (2)	1.000
**Respiratory failure**	3 (7)	1 (2)	0.616
**Sepsis**	3 (7)	6 (13)	0.482
**Pneumonia**	0	1 (2)	1.000
**Wound infection**	1 (2)	0	1.000
**Urinary tract infection**	0	3 (7)	0.240

Values in parentheses are percentages.

## Discussion

This double-blind randomized trial studied the effect of RIPC on cardiac and inflammatory biomarkers in patients undergoing pancreatic surgery and showed that RIPC did not reduce the maximum postoperative hs-cTnT concentration, modify postoperative concentrations of IL-6 or influence postoperative complications. All results were similar to those seen in control patients who had received a sham intervention.

The cardioprotective effect of RIPC is well established in patients undergoing cardiac surgery. Recent meta-analyses have shown that postoperative cardiac biomarker concentrations were lower in cardiac surgery patients after RIPC^11–13^. In non-cardiac surgery, a randomized trial with RIPC in abdominal surgery patients (more than 90 per cent of patients underwent colon surgery) found no differences in hs-cTnT concentrations until 72 hours after surgery^23^. Although pancreatic surgery is generally considered a higher risk than colonic surgery (i.e., longer duration, more blood loss, higher rate of postoperative complications), and more patients had PMI in the present study population (34 *versus* 21 per cent in the previous study), no reduction in cTnT concentrations was observed after RIPC at any time. Compared to cardiac surgery, peak cTn concentrations are 10 to 20 times lower than after major abdominal surgery. This may reflect the relatively low prevalence of coronary artery disease in the present cohort, where only 20 per cent of patients had established coronary artery disease (prior myocardial infarction or coronary revascularization). Baseline hs-cTnT of 14 ng/l or greater was present in one out of five patients of the total study population. Minimally invasive robotic surgery, epidural anaesthesia and restricted transfusion management may all have suppressed the ischaemia–reperfusion injury, targeted by RIPC. Possible interference of propofol with the cardioprotective effects of RIPC, which has been extensively described in prior studies, was ruled out by the present study design^11^^,^^13^.

Surgical tissue injury triggers pro- and anti-inflammatory pathways. Excessive activation of the immune system, reflected by high concentrations of IL-6, has been associated with adverse outcome after abdominal surgery^9^^,^^24^. Reports on the effect of RIPC on perioperative inflammation are essentially confined to patients having cardiac surgery. In 206 patients who underwent combined cardiac rhythm and valve surgery, RIPC significantly decreased C-reactive protein (CRP) and neutrophil–lymphocyte ratio (NLR)^25^. Similar results were found in a cohort of 72 patients receiving radiofrequency ablation for atrial fibrillation, where the RIPC group showed significantly attenuated increases in CRP and IL-6^26^. There are, nevertheless, other studies in patients undergoing major cardiac surgery, that have found no association between RIPC and postoperative concentrations of IL-6 and tumour necrosis factor-α,^28^. The perioperative immune response is complex and likely to differ between surgical procedures. Ischaemia–reperfusion injury in pancreatic surgery may not be as extensive as in cardiac surgery and could explain the lack of effect of RIPC on the perioperative inflammatory response in the present study.

The present study has several limitations. Postoperative concentrations of hs-cTnT were relatively low. This might be the result of the study design. Risk factors for coronary artery disease were not an inclusion criterion, in contrast to a previous study performed at this centre^1^. As fixed coronary artery disease is believed to play an important role in PMI, this may have influenced the results. The trial was not primarily designed to detect differences in IL-6 concentrations and postoperative complications and may have been underpowered in assessing these variables. Lack of an effect on these outcomes may be explained by a type two statistical error.

Cardiac and inflammatory biomarkers were not reduced by RIPC after pancreatic surgery and results were similar to those for control subjects who received a sham intervention. Future studies on the effect of RIPC in major non-cardiac surgery should focus on patients with significant cardiac disease.

## Funding

Funding for biomarker analysis was provided by Roche Diagnostics. Roche Diagnostics had no role in design and conduct of the study, analysis and interpretation of the data, or preparation and approval of the manuscript.

## Supplementary Material

zrab015_Supplementary_Data
